# Geopolymer Concretes with Organic Phase Change Materials—Analysis of Thermal Properties and Microstructure

**DOI:** 10.3390/ma18112557

**Published:** 2025-05-29

**Authors:** Agnieszka Przybek, Michał Łach, Paulina Romańska, Justyna Ciemnicka, Karol Prałat, Artur Koper

**Affiliations:** 1 CUT Doctoral School, Cracow University of Technology, Warszawska 24, 31-155 Cracow, Poland; 2 Faculty of Material Engineering and Physics, Cracow University of Technology, Jana Pawła II 37, 31-864 Cracow, Poland; paulina.romanska@pk.edu.pl; 3 Interdisciplinary Center for Circular Economy, Cracow University of Technology, Warszawska 24, 31-155 Cracow, Poland; 4 Faculty of Civil Engineering, Mechanics and Petrochemistry, Warsaw University of Technology, Łukasiewicza 17, 09-400 Płock, Poland; justyna.ciemnicka@pw.edu.pl (J.C.); karol.pralat@pw.edu.pl (K.P.); artur.koper@pw.edu.pl (A.K.)

**Keywords:** geopolymer concretes, organic phase change material (PCM), functional materials for energy saving, composites for modern building, thermal properties, microstructure

## Abstract

Geopolymer concretes, synthesized from industrial by-products such as fly ash through alkaline activation, have attracted considerable attention due to their favorable thermal and microstructural properties. Incorporating phase change materials (PCMs) into geopolymer matrices can improve thermal properties, making them suitable for various sustainable construction applications. The thermal properties of geopolymer concrete depend on the composition and structure of the materials used. Adding PCMs to geopolymer concrete can significantly improve its thermal properties by increasing its heat storage capacity. PCMs absorb and release thermal energy during phase transformations, which can help regulate temperature fluctuations in building materials. This feature is particularly beneficial in regions with extreme temperature fluctuations, where maintaining a stable indoor climate is crucial. Integrating organic PCMs into geopolymer matrices has been shown to improve thermal insulation. Furthermore, the microstructural analysis of geopolymer concrete containing organic PCM indicates that incorporating these materials can lead to a more homogeneous and denser microstructure. Integrating organic PCMs instead of inorganic into geopolymer concrete is a promising route to improve thermal properties and microstructural stability. The combination of geopolymer technology with PCM not only contributes to the sustainable development of building materials but also addresses the challenges of temperature regulation in buildings.

## 1. Introduction

### 1.1. Geopolymers—Benefits and Production from Waste Materials

Geopolymer concretes represent innovative building materials that offer a sustainable alternative to traditional Portland cement-based concretes. They are synthesized through the alkaline activation of aluminosilicate materials, such as fly ash, slag, or other industrial by-products, in combination with alkaline activators, including sodium hydroxide (NaOH) and sodium silicate [1,2,3]. This process results in the formation of a three-dimensional network of inorganic polymers that provides unique properties, including elevated compressive strength, durability, and resistance to various environmental factors [4,5,6].

One notable characteristic of geopolymer concretes lies in their mechanical properties. Research indicates that these materials demonstrate superior compressive strength compared to conventional concretes [7,8]. Numerous factors can influence the strength of geopolymer concretes, including the choice of precursor material, the molarity of the alkali activator, and the curing conditions [9,10,11]. For instance, investigations have revealed that activators with higher molarity (e.g., 16 M) can enhance the durability and mechanical properties of geopolymer concretes [12,13]. Furthermore, the incorporation of fibers, such as steel or plant fibers, can further augment the ductility and fracture resistance of geopolymer concretes, rendering them suitable for a variety of structural applications [14,15,16].

Another pivotal aspect of geopolymer concretes pertains to their environmental impact. Their production results in reduced carbon dioxide emissions in comparison to the traditional manufacturing processes associated with conventional concretes, primarily due to the utilization of industrial waste as precursors [17,18,19]. This not only contributes to sustainability within the construction industry but also aids in alleviating environmental issues related to waste disposal from sectors such as energy, fossil fuels, and metallurgy [20,21,22]. Moreover, geopolymer concretes exhibit exceptional resistance to chemical environments, fire, and extreme weather conditions, thus enhancing their durability and longevity in construction applications [23,24,25]. With respect to workability, geopolymer concretes can present challenges owing to their rapid setting time, which frequently exceeds that of conventional concretes [26,27,28]. This characteristic necessitates meticulous management to ensure the workability of the concretes until their application on site. Nevertheless, strategies such as incorporating retarders or optimizing the mix design can effectively mitigate this issue [29,30,31].

The characteristics of geopolymers contribute to their recognition as environmentally friendly alternatives to traditional cementitious materials in construction and other applications [32,33,34,35,36]. An important aspect is the ability to produce them from a diverse array of raw materials, including various kinds of industrial waste. Fly ash, a byproduct of coal combustion, has been widely studied and used as a precursor to synthesize geopolymers, complementing other sources such as metakaolin and slag [37,38,39]. Similarly, the incorporation of ground granulated blast-furnace slag (GGBS) alongside fly ash has been shown to improve mechanical properties, particularly when optimizing activator ratios, as evidenced by Deb et al. [40]. The combined use of GGBS and jarosite, a by-product of zinc production, was explored by Pandiyan and Elavenil [41], demonstrating the feasibility of utilizing multiple industrial wastes to develop geopolymer concrete with improved strength and durability. Kotsay and Grabowski [32] investigated the use of soda-lime waste glass in metakaolin-based geopolymers, emphasizing the potential to recycle glass waste effectively while enhancing mechanical performance. Moreover, components such as recycled fibers and aggregates, sawdust, and brick ballast have been explored for geopolymer formation [42]. These are only selected examples that underscore the versatility of geopolymers as a sustainable alternative for waste management and material innovation.

The formulation of geopolymers can be tailored to the desired properties and applications using different proportions of precursors and alkali activators. Optimal performance properties, such as the balance of mechanical and physical properties, can be achieved through precise parameter selection [43,44,45]. Ongoing research into the integration of bio-based and recycled materials in geopolymer formulations indicates a growing trend toward creating sustainable building materials that meet modern environmental standards [39,46]. The adaptability of geopolymers makes them a promising material for sustainable construction.

### 1.2. Organic PCMs—Properties and Use in Modifying Binders and Geopolymers

Organic phase change materials (PCMs) have attracted considerable attention due to their ability to efficiently store and release thermal energy through phase transitions, mainly from solid to liquid and vice versa. These materials, which include fatty acids, paraffin waxes, and polyethylene glycols, are particularly beneficial in building energy efficiency and thermal comfort applications. Their effectiveness is attributed to several key properties, including high latent heat, non-toxicity, and minimal volume change during phase transitions [47,48,49]. The thermal performance of organic PCMs is enhanced by their ability to absorb and release large amounts of energy at near-constant temperatures, making them ideal for indoor climate control in buildings. For example, studies have shown that PCMs can significantly reduce energy consumption by shifting heating and cooling loads from peak to off-peak periods, thereby improving the overall energy efficiency of HVAC systems [50,51]. Furthermore, integrating PCMs into the building envelope can stabilize indoor temperatures, providing a more comfortable living environment while reducing reliance on conventional energy sources [52].

Research has shown that selecting suitable organic PCMs is crucial for optimizing their performance in specific applications. For example, the melting point and heat of fusion of different organic materials can vary significantly, affecting their suitability for different climates and building designs [51,53]. The development of composite PCMs, which combine organic and inorganic materials as well as other additives, has been shown to improve thermal properties and expand the range of applications [53,54]. This approach allows for the fine-tuning of phase transition temperatures and latent heat capacity to better meet specific energy storage needs [53]. Encapsulation techniques are also essential for the practical application of organic PCMs, as they prevent leakage and increase thermal conductivity. Recent advances in nanocomposite materials, such as those containing carbon nanotubes or graphene, have shown promise in improving the heat transfer properties of PCMs, thereby increasing their performance in energy storage applications [55]. Hermitization not only protects the PCM but also facilitates its integration into various building materials, such as wallboard and plaster, further enhancing their thermal performance [56,57]. The further exploration of their applications will likely lead to more innovative solutions for energy conservation and thermal comfort in the built environment.

The integration of organic PCMs instead of inorganic ones into geopolymer concrete is gaining research interest and emerging as a promising approach to improve its thermal properties and energy efficiency [58,59,60,61]. Moreover, utilizing organic PCMs derived from renewable sources can further improve the overall sustainability profile of geopolymer concrete, making it a more appealing choice for green building practices [62]. Paraffin and bio-based PCMs have been shown to increase the specific heat capacity of geopolymer composites, enabling these materials to absorb and store thermal energy more efficiently, thereby optimizing their performance in thermo-regulating applications [63,64]. Based on computer simulations, Cao V.D. et al. demonstrated that integrating encapsulated MPC into multilayer geopolymer walls in a single dwelling in Oslo (Norway) significantly improved thermal performance, reducing annual energy consumption by 28–30% [65]. Additionally, Pilehvar et al. found that the addition of PCM to geopolymer concrete significantly enhances thermal storage capacity and increases resistance to freeze–thaw cycles, maintaining structural integrity under severe temperature fluctuations [66,67,68]. Studies indicate that the mechanical properties of geopolymer concrete can be maintained or even improved by adding organic PCMs. As indicated in our own research, the incorporation of form-stable PCMs into geopolymer mortars has demonstrated significant improvements in thermal properties while maintaining adequate mechanical strength, although some reduction in compressive strength has been observed [69,70]. Additionally, it has been demonstrated by various authors that the compressive strength of geopolymer concretes is comparable to that of conventional concrete, even following the incorporation of PCMs [71,72]. Furthermore, research has underscored the potential of utilizing PCM-impregnated lightweight aggregates to enhance the insulating properties and thermal energy storage capacity of geopolymer composites [73,74]. Lightweight expanded clay aggregates (LECAs), which have been post-coated with geopolymer paste, have been effectively employed to develop macro-encapsulations of PCMs, thereby augmenting the structural integrity of the material during phase transition cycles [74].

Despite several promising findings, the number of studies regarding geopolymers with organic PCMs remains relatively limited, and the existing literature lacks a systematic and comprehensive approach.

### 1.3. Balancing Thermal Functionality and Structural Integrity

Interfacial interactions between the PCM and the geopolymer matrix are crucial for determining the overall performance of the composite, while encapsulation techniques are essential for maximizing the retention efficiency and functionality of the PCM [75,76]. The key is the careful selection of PCM types and their proportions in the geopolymer matrix. This balance ensures that the favorable thermal properties of PCMs do not compromise the structural integrity of the concrete [77]. Optimal formulations range from 5% to 20% PCM by weight [64,70].

The morphology of PCMs, particularly the differences between microcapsules and macrocapsules, has a significant impact on the thermal properties and overall performance of geopolymer composites. Understanding these differences is key to optimizing the incorporation of PCMs into geopolymer matrices, especially in applications requiring temperature management. Microcapsules tend to be smaller (from a few micrometers to about 100 μm) and can exhibit increased thermal conductivity compared to larger macrocapsules. For example, a study by Yoo et al. indicates that the encapsulation of PCMs in microcapsules can significantly improve heat transfer mechanisms due to their larger surface-to-volume ratio, which facilitates efficient heat transfer between the PCM core and the surrounding matrix [78]. This property is particularly important for geopolymers, as the higher thermal conductivity helps with rapid heating and cooling, enabling better thermal response in applications such as building materials. Macrocapsules, on the other hand, which are typically larger than 100 μm and can be up to a few millimeters in diameter, can provide benefits related to structural integrity and ease of handling, but can hinder heat transfer rates. As Kong et al. pointed out, the performance of macrocapsules is affected by their thermal conductivity and the design of their walls, which must be optimized to minimize thermal resistance and maximize heat transfer efficiency [79]. The thickness and material of capsule walls can create barriers to efficient heat transfer, leading to slower phase transformation reactions compared to microencapsulated systems.

The encapsulation morphology also affects the dynamics of PCM phase transitions. In the case of microencapsulated PCMs, a thinner shell allows for rapid heat uptake and release, which is crucial for energy storage applications. Latibari et al. showed that microencapsulated PCMs can exhibit a higher heat transfer coefficient due to increased surface exposure [80]. As a result, microcapsules tend to respond more quickly to temperature fluctuations, making them suitable for applications where rapid temperature control is necessary. In contrast, macrocapsules, although they can accommodate larger volumes of PCMs, can experience slower phase change dynamics. This can be attributed to the greater thermal inertia associated with the larger mass and potentially thicker walls, resulting in delayed thermal responses, as noted in the literature on closed systems [81]. The integration of microcapsules into geopolymer matrices can lead to improved thermal stability and energy storage capacity without the significant degradation of mechanical properties. Studies have shown that the incorporation of PCM microcapsules increases the thermal permeability of the geopolymer composite, which can improve energy efficiency in construction applications [82]. In addition, the rheological properties of geopolymer pastes can also be affected by the type of encapsulation, as indicated by the work conducted by Cao et al. who showed that microencapsulated PCMs had a particular effect on the viscosity and workability of geopolymer mixtures, facilitating mixing and resulting in a more homogeneous composite [83]. This serves to highlight the practical advantages of microcapsules in producing samples with consistent thermal properties. In summary, microcapsules typically provide enhanced thermal conductivity, accelerated phase transformation reactions, and beneficial impacts on the rheological properties of geopolymer formulations. Conversely, macrocapsules, while beneficial due to their structural integrity and greater capacity, present challenges regarding thermal performance and reaction time. Therefore, optimizing the type of encapsulation used in geopolymer composites is essential to maximize their thermal properties and practical application in thermal energy storage and regulation.

The practical application of geopolymer concrete with organic PCMs presents several challenges. The compatibility of PCMs with the alkaline environment of geopolymer concrete must be carefully assessed to prevent non-corrosive reactions that could affect the durability and performance of the material [84]. Additionally, the long-term stability of PCM in a geopolymer matrix under varying environmental conditions is an area that requires further research [85].

The objective of this study was to analyze the physicochemical and thermal properties of geopolymer concretes with organic PCMs and, most importantly, to evaluate the energy efficiency of these composites. The compatibility of PCMs with the geopolymer matrix was also assessed, along with the applicability of this technology in energy-efficient construction. To date, the majority of research has predominantly focused on the utilization of inorganic PCMs, specifically hydrated salts. In contrast, this study examined organic paraffinic PCMs, which are characterized by more stable thermal properties and prolonged service life. The research undertaken was motivated by the absence of comprehensive studies regarding the impact of organic PCMs (i.e., paraffinic PCMs) on the properties of geopolymer concretes, particularly concerning component compatibility, thermal stability, and energy efficiency. Therefore, it was considered essential to investigate the extent to which organic PCMs can be effectively integrated into the geopolymer matrix and whether they can enhance the material’s thermal performance in the context of energy-efficient construction applications. This work holds both scientific and practical significance. It provides data that may be useful in the further development of building materials with superior thermal performance. It is expected that the combination of geopolymers (which can be produced from industrial waste, such as fly ash) with organic PCMs presents a novel and environmentally sustainable alternative to conventional building materials. This integration should facilitate increased thermal capacity and stability, resulting in reduced temperature fluctuations within indoor environments. Consequently, it may contribute to a decrease in the energy requirements for heating and cooling in energy-efficient buildings.

## 2. Materials and Methods

### 2.1. Raw Materials for the Manufacture of Geopolymer Concretes

The geopolymer concretes incorporated F-class fly ash derived from coal combustion at the Skawina Combined Heat and Power Plant (CEZ Skawina S.A., Skawina, Poland), quartz sand from the Świętochłowice Sand Plant (Świętochłowice, Poland), and high alumina cement with the trade name Górkal 70 (Górka Cement, Trzebinia, Poland) as a hydraulic additive. Additionally, the concretes utilized ash microspheres (TERMO-REX S.A., Jaworzno, Poland) and the surfactant Syringaldehyde (Merck, Germany). The concretes were produced in a manner identical to that of the geopolymer foams examined in prior studies conducted by the authors [70,86,87]. The only modification was the absence of a foaming agent. Chemical composition analysis by XRF fluorescence was performed for fly ash, sand, cement, and microspheres on a SCHIMADZU EDX-7200 (SHIMADZU Europa GmbH, Duisburg, Germany), with the results shown in Table 1. Laserial particle size analysis was also performed on an Anton-Paar PSA 1190LD (Anton Paar GmbH, Graz, Austria), and the results are presented in Table 2.

### 2.2. Phase Change Materials

**Figure 1:**
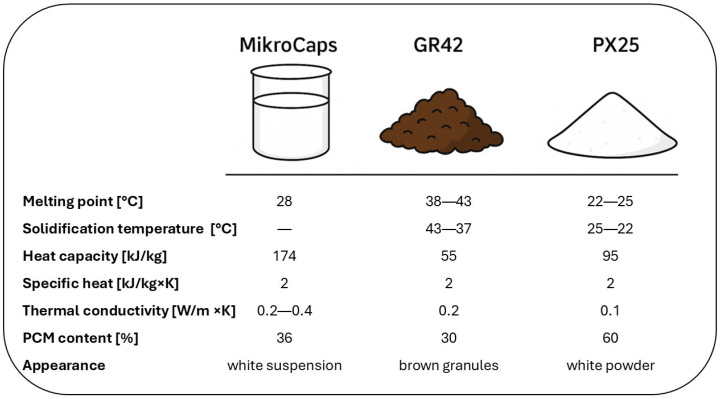
PCMs used in geopolymer concretes [88,89].

Three different organic, paraffinic PCMs were introduced into the composition of geopolymer concretes to study their impact on the properties of the composite. These materials exhibited varying phase transition temperatures ranging from 22 °C to 42 °C. In addition to differing thermal parameters, the materials also varied in their physical form. The first material, MikroCaps PCM 28 Slurry (MikroCaps, Ljubljana, Slovenia), was a suspension of microcapsules containing paraffin. The other two products, GR42 and PX25, supplied by Rubitherm (Berlin, Germany), were macrocapsules with paraffinic PCM inside. The detailed characteristics of the PCMs used are summarized in Figure 1. Table 3 shows particle size measurements for the PCMs.

### 2.3. Preparations of Geopolymer Concretes

**Figure 2:**
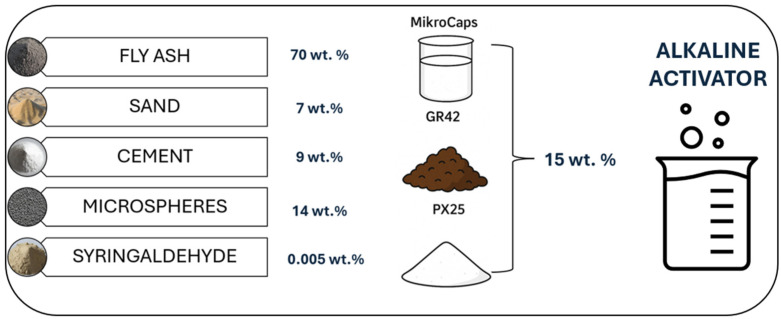
Composition of ready-made geopolymer concretes with PCMs.

A composition of raw materials, including F-grade fly ash, quartz sand, high alumina cement, ash-derived microspheres, and a surfactant in the form of Syringaldehyde, was utilized in the production of geopolymer concretes. Sand and microspheres functioned as filler materials, thereby influencing matrix compaction and modifying the rheological properties of the fresh mixture. The initiation of the geopolymerization reaction was conducted with a 10 mol solution of alkaline sodium silicate, prepared using sodium water glass. Sodium silicate R-145, characterized by a molar modulus of 2.5 and a density of 1.45 g/cm^3^, obtained from the ANSER Chemical Plant (Wiskitki, Poland), was combined with caustic soda in flake form (PCC Group, Brzeg Dolny, Poland). In order to assess the impact of PCMs on the functional properties of the geopolymers, 15 wt.% PCM additives were incorporated into the mixture. This quantity was selected as the maximum technically feasible to introduce while preserving the structural integrity of the material. The PCMs employed contained between 30 wt.% and 60 wt.% of active paraffinic material within their composition, with the objective of enhancing thermal insulation properties and augmenting heat storage capacity. It is notable that the presence of PCM within the structure of a geopolymer can influence not only its thermal properties but also its hydration process and microstructure, particularly in terms of the compatibility of the phase-change material with the inorganic binder matrix. Potential effects encompass improved thermal comfort in building applications and the feasibility of utilizing geopolymers as thermal energy storage elements in smart energy management systems. The detailed composition of all the samples produced is summarized in Figure 2.

The procedure for producing geopolymer concrete specimens with the addition of PCMs began with the homogenization of the dry components, namely fly ash, high alumina cement, quartz sand, ash microspheres, and a surfactant. The mixing process was conducted in a laboratory mixer of the M/LMB-s type (GEOLAB, Warsaw, Poland) for approximately five minutes at a speed of 58 revolutions per minute. Subsequently, GR42 and PX25 PCMs were integrated into the resultant mixture, added directly to the solid phase. For the MikroCaps material, due to its peculiarities (presented as a suspension of microcapsules), the additive was introduced only following the initial mixing of the dry components. The next step involved the addition of an alkali activator in the form of a 10 molar solution of sodium silicate with water glass, which initiated the geopolymerization process. The complete mixture was stirred for an additional ten minutes until a homogeneous, plastic mass was achieved. In geopolymers containing PCMs, particularly organic ones (e.g., paraffinic), one significant challenge is the aggregation of PCM particles. This aggregation results in uneven distribution of the PCM in the matrix, a reduction in the mechanical properties of the composite, and a decline in heat storage efficiency. To mitigate this issue, various means and methods have been employed. For these composites, the dispersing agent surfactant Syringaldehyde was utilized, facilitating the uniform distribution of PCM throughout the matrix. MikroCaps comprises PCM encapsulated in microcapsules, which inhibits leakage during phase transitions, enhances compatibility with the matrix, reduces agglomeration, and aids in dispersion. Additives such as GR42 and PX25 are incorporated into porous materials (silica), thereby preventing PCM mobility and agglomeration. The process also incorporates an optimized mixing procedure. The correct sequence for introducing ingredients, along with meticulous control of mixing time and intensity, significantly minimized agglomeration. The finalized material was transferred into molds, subsequently undergoing an annealing process at 60 °C for 24 h in a SLW 750 laboratory dryer (POL-EKO Perfect-Environment, Wodzisław Śląski, Poland). The curing temperature of geopolymers is crucial in determining the physicochemical properties of the composite, particularly in the presence of PCMs within the matrix. Nevertheless, elevated temperatures (for instance, above 60–80 °C), often used to accelerate the geopolymerization reaction, can adversely affect the thermal stability of encapsulated PCMs, especially those of organic nature, such as paraffins. Research indicates that excessively high curing temperatures may inflict damage upon microcapsules, resulting in PCM leakage, the degradation of their properties, and a disruption of the material’s structural integrity. Moreover, the curing temperature may influence the phase transition temperature of the PCM, leading to shifts, changing the enthalpy during phase transitions or a reduction in heat storage capacity. Furthermore, at elevated temperatures, undesirable chemical or physical reactions may manifest at the geopolymer-PCM interface, compromising adhesion and engendering defects within the transition zone. The literature indicates that the optimal curing temperature for microencapsulated PCMs should be maintained within the range of 40–60 °C, thereby preserving the stability of the microcapsules and minimizing the risk of damage. Consequently, when designing geopolymers with incorporated PCMs, it is imperative to achieve a balance between the efficacy of the curing process and the preservation of PCM functionality and interface stability. Additionally, consideration must be given to the type of capsule casing and the composition of the PCM itself [90,91,92].

**Figure 3:**
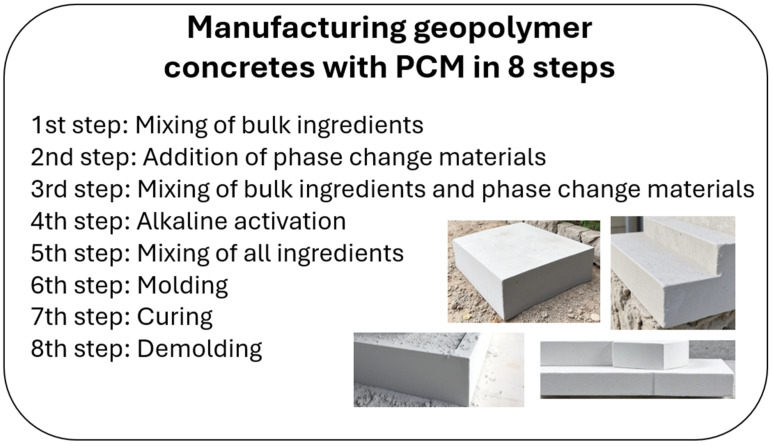
Scheme of manufacturing geopolymer concretes with PCMs.

Following the curing stage, the samples were extracted from the molds and set aside for further testing. All samples were produced in the form of cubes measuring 10 cm × 10 cm × 10 cm, facilitating the standardized analysis of thermal properties. The technological process is depicted in the form of a diagram in Figure 3, illustrating the sequential stages involved in the production of PCM-modified geopolymers. The utilization of microspheres and surfactants not only modifies the porous structure but also enhances the stability of the suspension and reduces component segregation, a factor of particular importance in the presence of PCMs. Furthermore, appropriate annealing conditions during the initial curing phase support both effective alkaline activation and the stabilization of PCMs within the geopolymer matrix. The designation established for the reference sample was F.A.—ref, and for samples containing 15 wt.% PCM content, the following designations were adopted: 15 wt.% MC, 15 wt.% GR42, and 15 wt.% PX25 (where MC denotes MikroCaps).

### 2.4. Density Measurements

The bulk density of the hardened geopolymer concrete samples was determined by the geometric method, which involves calculating the ratio of mass to the known volume of solids. All samples possessed identical nominal dimensions of 10 cm × 10 cm × 10 cm, correlating to a volume of 1000 cm^3^; however, their mass exhibited slight variations contingent upon the composition of the mixture, likely attributable to the distribution of ingredients within the matrix. The precise measurements of the dimensions were conducted using a laboratory caliper, ensuring a precision of 0.01 mm, which enabled the detection of even minimal deviations from the design dimensions. The weight of the samples was determined using an analytical balance, RADWAG PS 200/2000, characterized by an accuracy of 0.01 g, thus guaranteeing the high reliability of the obtained results. The measurement of density constitutes a critical parameter in evaluating the quality of geopolymer materials, as it directly influences their insulating and thermal properties. In instances where the samples incorporate PCMs, variations in density may also serve as an indicator of the degree of PCM integration within the matrix and potential concerns regarding the homogeneity of the mixture. Consequently, density analysis serves as a valuable diagnostic instrument during the initial assessment of a material’s functional properties.

Knowing the exact geometric dimensions and mass of each sample, their volumetric density (denoted as *ρ*_b1*b1*_) was calculated according to the classical relation (1):

(1) $$\rho_{b1}=\frac{m}{V}\;[\frac{kg}{m^{3}}]$$

where *m* is mass and *V* is volume.

In addition, the measured thermal parameters allowed us to calculate the density *ρ*_b2_ from relation (2):

(2) $$\rho_{b2}=\frac{\lambda }{{\alpha C}_{p}}[\frac{kg}{m^{3}}]$$

where λ is thermal conductivity, α is thermal diffusivity, and *C_p_* is specific heat.

### 2.5. Procedure for Water Leachability Testing

The subsequent phase in the evaluation of the properties of the prepared geopolymer materials involved conducting leachability testing, which was assigned to the specialized laboratory of AP Geotechnika located in Siemianowice Śląskie, Poland. This entity offers a comprehensive range of services in the realm of physicochemical analysis of both natural and anthropogenic aggregates, industrial waste, soils, and construction materials, in accordance with the relevant national and European standards. For the purpose of testing, each sample underwent a grinding process utilizing a ball drum mill (model C20-ABLA-1, manufacturer: ATEST, Kielce, Poland) until a powdered form was achieved. A sample weighing 500 g of the crushed material from each series was prepared, enabling analysis under conditions that adhered to standard laboratory procedures. Leachability tests represent a critical component of the environmental assessment of construction materials. In the context of geopolymers, such tests facilitate the determination of the degree of immobilization of potentially harmful elements or compounds, including heavy metals, byproducts from alkaline reactions, or constituents of PCMs. Consequently, the results from leachability testing provide essential information regarding the environmental safety of the material and its appropriateness for sustainable construction or applications in environmental engineering.

### 2.6. Acquiring λ, C_V_, C_p_, and α of Geopolymer Concretes with PCMs

Tests assessing the thermal properties of the prepared samples were conducted at the Department of Civil Engineering, Mechanics and Petrochemistry of Warsaw University of Technology in Plock, Poland. The measurements were performed following the procedure outlined in ASTM D5334-08, utilizing a microprocessor-based Isomet 2114 measuring device (Applied Precision Ltd., Bratislava, Slovakia). Six independent measurements were executed for each sample, yielding statistically averaged results characterized by high reliability. The Isomet 2114 serves as an advanced thermal property analyzer employing the heat pulse method. During the measurement process, a known heat source is generated, which propagates radiatively within the material under examination. This heat flows through a probe in direct contact with the sample, enabling the recording of temperature change data over time through semiconductor sensors positioned at specific points. The analyzer computes the thermal conductivity (λ) and volumetric specific heat (*C*_V_) based on the linear correlation of temperature increase against the logarithm of time. In the study presented herein, a surface probe was utilized, specifically designed for measuring materials with greater hardness, such as geopolymers. The Isomet 2114 provides a broad measurement range: thermal conductivity spanning from 0.015 to 6.0 W/m × K and volumetric specific heat ranging from 0.04 to 3 MJ/m^3^ × K, while ensuring a measurement accuracy of ±5%. The recorded data can be stored directly in the device’s memory or exported to a computer system via a serial port (RS-232C) for subsequent analysis and archiving. The application of the pulsed method to measure thermal conductivity represents a significant advantage in studying heterogeneous materials, such as geopolymers modified with PCMs. This technique is distinguished by its non-invasiveness and high sensitivity, thereby facilitating the accurate capture of the impact of PCMs on heat transport within a geopolymer matrix. The precise determination of λ enables the assessment of the insulating potential of the tested materials, which is crucial in the context of their application in energy-efficient construction and thermal energy management systems.

According to the second law of thermodynamics, thermal conductivity (λ) is determined by an equation derived from Fourier’s law, which describes the linear flow of heat in solid media. For the pulse method used in the study, with the heat flux known and the temperature change measured as a function of time, this relationship takes the following form (3):

(3) $$\;\lambda =\frac{Qd}{A∆T}[\frac{W}{m\times K}]$$

where *Q* is the amount of heat transferred, *d* is the distance between two isotherms, *A* is the area, and ∆*T* is the temperature gradient.

In the context of the Isomet 2114 device employed, the analysis is based on a simplified model of heat conduction within a semi-infinite medium, wherein the temperature distribution surrounding the heat probe exhibits logarithmic variation over time (*t*) (4):

(4) $$∆T=f(ln\left(t\right))$$

This equation enables the direct determination of λ without relying on classical stationary methods. It aligns with the principles of thermodynamics, as it depicts heat flow as an irreversible process that aims to achieve thermal equilibrium, in accordance with the second principle of thermodynamics.

Volumetric heat capacity (*C_V_*) constitutes a significant thermophysical parameter that determines a material’s capacity to store thermal energy. It quantitatively represents the amount of heat required to elevate the temperature of a unit volume of material by one kelvin (1 K). This particular quantity holds substantial importance within the framework of the analysis of PCMs and other materials, including geopolymers. The value of *C_V_* is determined utilizing Equation (5):

(5) $$C_{V}=\frac{Q}{V_{c}∆T}=C_{p}\times \rho_{b1}[\frac{kJ}{m^{3}\times K}]$$

where *Q* is the amount of heat transferred, *V*_c_ is volume, ∆*T* is temperature gradient, *C*_p_ is specific heat, and *ρ*_b1_ is density.

Specific heat capacity (*C_p_*), also known as specific heat, is the basic thermodynamic parameter describing a substance’s ability to store thermal energy. It is defined as the amount of heat required to raise the temperature of 1 g (or 1 kg, depending on the units) of a substance by 1 kelvin (1 K), at constant pressure. The value of *C_p_* can be expressed using Equation (6):

(6) $$\;C_{p}=\frac{Q}{m∆T}=[\frac{J}{kg\times K}]$$

where *Q* is the amount of heat transferred, *m* is mass, and ∆*T* is temperature gradient.

Thermal diffusivity (α) is a property of a material that determines the rate of heat transfer from an area of higher temperature to an area of lower temperature. It is an indicator of the rate of propagation of heat waves in a material. Thermal diffusivity is crucial in materials that are used for heat management (7):

(7) $$\alpha =\frac{\lambda }{\rho_{b1}C_{p}}=[\frac{{mm}^{2}}{s}]$$

where λ is thermal conductivity, *ρ*_b1_ is density, and *C_p_* is specific heat.

The thermal properties were measured for all modified geopolymer samples incorporating PCMs. Six independent measurement runs were conducted for each sample to ensure the repeatability and reliability of the results. The analysis included three fundamental thermal parameters, namely thermal conductivity (λ), volumetric heat capacity (*C_V_*), and thermal diffusivity (α), which collectively represent a fundamental set of characteristics necessary for evaluating a material’s capacity to conduct, store, and disseminate heat. Additionally, based on the acquired values of *C_V_* and the material’s volumetric density (*ρ*_b1_), the specific heat (*C_p_*) was subsequently determined.

When the standard deviation of a random variable X is unknown, and the goal is to estimate the expected value µ from the sample mean $\bar{X}$ Student’s t-distribution serves as an approximation for the distribution of this mean. This is especially true when the random variable X has a normal distribution N(μ,σ), while the population standard deviation σ remains unknown. Instead of the normal distribution, which requires knowledge of the value of σ, Student’s t-distribution with n − 1 degrees of freedom is utilized, where n represents the number of observations within the sample. The probability density of Student’s t-distribution is articulated through Formula (8):

(8) $$f\left(t,\;n\right)=\frac{\Gamma \left(\frac{n+1}{2}\right)}{\Gamma \left(\frac{n}{2}\right)\sqrt{n\pi }}{\left(1+\frac{t^{2}}{n}\right)}^{-\frac{n+1}{2}}$$

After appropriate statistical transformations, while considering the unknown population standard deviation and employing Student’s t-distribution, we ultimately derive the formula for the confidence interval for the unknown population mean (9):

(9) $$P\left(\bar{X}{-t}_{n-1;\alpha /2}\frac{s}{\sqrt{n}}\leq \mu \leq \bar{X}+t_{n-1;\alpha /2}\frac{s}{\sqrt{n}}\right)=1-\alpha$$

where α is the assumed significance level and 1 − α is the confidence level.

### 2.7. Microscopic Observations

A scanning electron microscope (SEM) model JEOL IT200 (JEOL Ltd., Akishima, Tokyo, Japan) was used to analyze the microstructural morphology of the fabricated geopolymer foams. Prior to observation, the samples were carefully prepared in accordance with established procedures that ensured the preservation of their structural integrity and the characteristics of PCMs. Sample fragments underwent pre-cleaning to eliminate residual dust and particles that could potentially interfere with surface analysis. Subsequently, they were dried at a controlled temperature of 40 °C, which allowed the removal of moisture while preventing detrimental physicochemical changes in the structure of PCMs, which are particularly sensitive to elevated temperatures. This process ensured that a constant weight of the samples was achieved, which is essential for the reproducibility of the tests. Carbon disks were employed to mount the samples, which were placed on metal preparation tables, all securely installed within a microscope holder. To enhance electron conductivity, EM-Tec C33 conductive carbon adhesive was utilized to promote proper bonding between the sample and substrate, thereby minimizing charge effects. In order to enable the effective imaging of non-conductive materials, the surface of each sample was coated with a thin layer of gold through vacuum sputtering, utilizing a DII-29030SCTR Smart Coater (JEOL Ltd., Peabody, MA, USA). This metallic coating significantly enhances the quality of imaging by eliminating the phenomenon of electrical charge buildup on the surface of the sample, commonly referred to as “charging”. The application of SEM in the analysis of geopolymer concretes facilitates the assessment of structural homogeneity and the identification of microcracks or the distribution of PCMs within the matrix. This methodology is pivotal in materials engineering, enabling the correlation of microstructural parameters with the physical and thermal properties of geopolymer composites.

## 3. Results

### 3.1. Density of Geopolymer Concretes with PCMs

Four types of geopolymer samples were prepared: a reference sample (containing no phase PCM) and three samples incorporating 15 wt.% PCM, differing in the type of PCM utilized. Each composite variant was replicated in four independent batches to average the results and enhance the reliability of the analysis. All samples were standardized to geometric dimensions of 100 mm × 100 mm × 100 mm, facilitating the comparability of the physical property data obtained. To ensure the accuracy of the results, six independent measurements were conducted for each sample. The average values of the results obtained are summarized in Table 4, which also illustrates a comparison of the volumetric density, denoted as *ρ*_b1_ (determined via the geometric method) and *ρ*_b2_ (calculated indirectly from thermal data), for all variants of geopolymer concretes. This approach enables the assessment of the impact of the type and presence of PCMs on the physical parameters of the composite material, which is critical for its subsequent engineering applications, particularly in the context of thermal energy storage and enhancing the energy efficiency of buildings. Furthermore, the comparison of *ρ*_b1_ and *ρ*_b2_ values provides insights into the consistency of the results obtained through different methods, thus contributing to the validation of the adopted research methodology.

Based on the measured analyses, it was observed that each of the applied PCMs exerted an influence on the reduction in the density of the tested geopolymer samples, as assessed through both geometric methods and calculations derived from thermal properties. The most significant decrease in *ρ*_b1_ and *ρ*_b2_ values, amounting to 13% for both parameters, was noted for the sample containing 15 wt.% MikroCaps, which exhibited the most pronounced effect among all tested variants. Conversely, the incorporation of GR42 resulted in a 3% reduction in density for both density measurements, while PX25 induced a 9% reduction in *ρ*_b1_ and *ρ*_b2_ values relative to the reference sample. These changes are substantial from an engineering perspective, given that PCM additives are expected to enhance density as a result of the introduction of additional mass into the concrete matrix. The findings imply that these effects may stem from variations in the consistency of the fresh mix, affecting the way the solids are compacted and distributed in the composite structure. The observed decrease in density may also correlate with modifications in the rheology and surface tension of the mixture, leading to altered structural organization during the setting and hardening processes. Notably, the sample with the MikroCaps additive—supplied as a liquid suspension of microcapsules—showed the most pronounced decrease in density, likely due to its effect on the homogeneity of the structure and the dilution of the geopolymer matrix. PCMs not only function as heat storage materials but also considerably affect the technological properties of geopolymer concretes.

### 3.2. Water Leachability of Geopolymer Concretes with PCMs

Table 5 provides a summary of the results from the analysis of the concentrations of selected potentially harmful substances present in leachates derived from the geopolymer concretes tested. It includes both the empirical values obtained and the corresponding limits set forth by current environmental regulations. Additionally, measurements of the pH of the samples and the content of chromium in its hexavalent form [Cr(VI)], recognized for its high toxicity and bioaccumulation capacity, are included in this summary. The analysis of heavy metals, chloride, fluoride, and sulfate ions, in addition to the presence of chromium (VI), was conducted in accordance with the requirements specified in ISO/IEC 17025, utilizing internal methodologies employed by the testing laboratory. It is worth noting that the ISO 17025 standard guarantees a high level of reliability and reproducibility of the analytical results obtained, which serves as the foundation for assessing the environmental safety of the materials tested. In parallel, the additional physicochemical parameters of the leachate were determined, which encompass moisture content, total dissolved solids (TDSs), and dissolved organic carbon (DOC). Although these indicators were analyzed using non-accredited internal procedures, they furnish valuable insights into the potential impact of geopolymer concretes on the quality of groundwater and surface water. All toxicological and chemical parameters, except for pH, were determined in the sample in the liquid state (in the so-called leachate), whereas the pH value was measured following the drying of the sample, which allows for a more representative assessment of the actual usage conditions of the material. This approach enables a comprehensive evaluation of the mobility of contaminants and their potential environmental impact under both operational and landfill conditions. The results are presented for two selected concrete samples to illustrate the concentration of harmful substances.

The table summarizes the permissible leaching levels for selected contaminants, in accordance with current landfill regulations. The base material used to obtain geopolymers was fly ash, classified as inert waste. Nevertheless, due to the incorporation of alkaline solutions (e.g., NaOH) as activators in the geopolymerization process, the final product must be classified as non-hazardous and inert waste. An analysis of compliance with the criteria for the disposal of hazardous waste in designated landfills revealed that all tested samples exhibit leaching levels of individual elements significantly below the permissible values. This indicates that, from an environmental safety perspective, the produced geopolymers do not display the toxic properties typically associated with hazardous waste. All analyzed samples demonstrated an alkaline reaction (pH > 10), which results directly from the chemical activation facilitated by the addition of alkaline solutions. This phenomenon promotes the initiation of polycondensation reactions, resulting in the formation of a stable geopolymer structure; however, it may simultaneously increase the mobility of certain elements, including heavy metals, as evidenced by the leachability test results. In accordance with the regulations currently in effect in Poland, waste intended for landfilling must adhere to specific limits, as indicated in the accompanying classification table. Accordingly, leachability tests were conducted to assess the potential environmental impact of these materials and to ascertain their waste classification. The observed exceedances of characteristic values for inert waste in some instances may arise from the presence of alkaline compounds, which may facilitate the leaching of constituents from fly ash, including heavy metals. Therefore, in the context of industrial utilization of this type of geopolymer material, it may be necessary to adopt additional purification steps for the final product, such as leaching or washing, to diminish the quantity of potentially harmful compounds and ensure adherence to environmental requirements.

### 3.3. λ, C_V_, C_p_, and α of Geopolymer Concretes with PCMs

Table 6 summarizes the detailed results of the thermal properties tests. The measurement data provided below represent the average values derived from four measurements for a specific sample variant (F.A.—ref.; 15 wt.% MC; 15 wt.% GR42; 15 wt.% PX25). The dimensions of the test specimens were consistent with those used in the density tests conducted at 100 mm × 100 mm × 100 mm. The mean values and standard deviations were documented for each sample, and these findings are presented in Table 7 to facilitate a clearer interpretation of the results.

Based on the *n* measurements carried out, the basic statistical characteristics of the thermal variables were determined, such as the arithmetic mean $\bar{X\;}$, and the standard deviation *s* calculated for the sample. On this basis, an approximation was established for the ranges within which the actual values of the measured parameters—namely thermal conductivity, specific heat, and thermal diffusivity—are expected to fall with a certain probability. For each of the cases analyzed, an estimation of measurement uncertainty was implemented using Student’s t-distribution, thereby facilitating the determination of confidence intervals for the specified thermal properties of the material. With a confidence level set at 95%, boundaries were delineated where the actual values of the thermal properties of the modified geopolymers are likely to be found with a high probability (approaching unity). These results are summarized in Table 8, which enables the comparative analysis and assessment of the impact of modification on the stability and homogeneity of the thermal parameters of the tested composites.

Based on the data presented regarding the thermal properties of geopolymer concretes modified with the addition of PCMs, it is evident that each of the components utilized has influenced the parameters of thermal conductivity, specific heat, and thermal diffusivity to varying extents. Significant differences were observed in the results of thermal conductivity (λ) when compared to the reference sample (1.1619 W/m × K). The inclusion of MikroCaps (MC) resulted in a reduction in this parameter by 22.2%, signifying a notable decrease in the material’s ability to conduct heat. Conversely, the introduction of GR42 resulted in a smaller yet still notable decrease of 4.6%. In contrast, the PX25 material exhibited an increase in thermal conductivity by 13.2% relative to the base sample, which may reflect the differing micromolecular structure and conductivity characteristics of the PCM in both solid and liquid states. Regarding volumetric specific heat, only the sample incorporating PX25 demonstrated a slight increase in this value (by 0.5%), whereas the other additives—MC and GR42—caused decreases of 2.0% and 4.7%, respectively. These findings suggest that the impact of PCMs on the material’s capacity to accumulate energy per unit volume is constrained and significantly reliant on the nature of the additive itself. In terms of specific heat, however, a clear increase in this parameter was evident in samples containing MC and PX25, with increases of 12.3% and 10.1%, respectively. This trend is beneficial from the perspective of designing heat-accumulating materials. The sample with GR42 experienced a slight decrease in specific heat (*C_p_*), amounting to 2.1%. Thermal diffusivity α, which describes the rate of heat propagation within the material, decreased by 20.7% in the sample with MC, indicating its diminished capacity to respond swiftly to temperature fluctuations. GR42 exhibited virtually no effect on this parameter (with a change in less than 1%), while PX25 contributed to an increase of 12.4%, highlighting its potential for applications necessitating rapid heat transfer. The additives MC and PX25 exert significant but opposing effects on thermal conduction and thermal storage properties. MikroCaps effectively reduce thermal conductivity and diffusivity while enhancing heat storage capacity, whereas PX25, by increasing λ, *C_p_*, and α, may be well-suited for materials where both rapid responsiveness to temperature changes and heat absorption capabilities are essential. The variance observed in these results underscores the necessity for the individualized selection of the type of PCM based on the intended function of the geopolymer concrete within the scope of thermal energy management. All tested samples reached a confidence level of 95%, indicating that 95% of the results fall within the range of actual values. The established confidence intervals align with the data summarized in Table 5 and Table 6.

### 3.4. Microstructure of Geopolymer Concretes with PCMs

**Figure 4:**
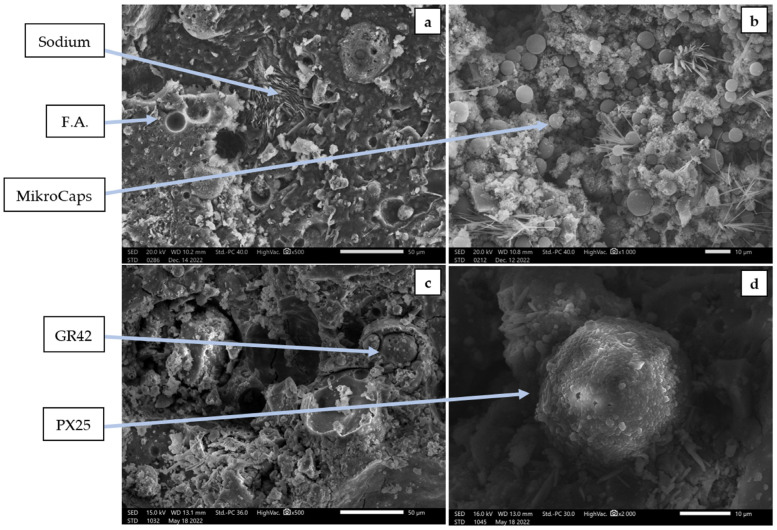
Microstructure of the geopolymer concretes with PCMs: (**a**) F.A.—ref.; (**b**) 15 wt.% MC; (**c**) 15 wt.% GR42; (**d**) 15 wt.% PX25.

Figure 4 shows the morphology of the structures of geopolymer concretes with the addition of PCMs. The images were taken at high magnifications to illustrate the PCMs contained in the samples, as well as their high cohesion with the geopolymer matrix.

SEM images illustrate the morphology of fly ash-based geopolymers in both their pure state and those modified through the incorporation of PCMs. The amorphous structure visible on the micrographs indicates a characteristic disordered geopolymer matrix, which is typical of alkali-activated materials. Arrows present in the images draw attention to unreacted fly ash particles as well as the inclusion of undissolved sodium particles and paraffinic PCM micro-additives. These elements are distinctly observable in areas of high magnification. An analysis of the microstructure demonstrates a favorable compatibility between the paraffinic particles and the geopolymer matrix; they are integrated within the material’s network in a manner that suggests robust physicochemical bonding. This observation indicates that the microcapsules not only preserve their structural integrity but also infiltrate the geopolymer matrix, enhancing heat transfer and storage within the material’s microstructure. The most significant presence and homogeneous distribution of particles were noted in the sample incorporating MikroCaps. Their even dispersion throughout the material’s volume implies a substantial degree of mixture homogenization and potentially an improved capacity for heat accumulation. Conversely, in samples containing GR42 and PX25 additives, the identification of PCM particles appears more challenging due to their larger size and reduced presence. Although they are indeed present, their distribution is less regular, which may influence the local inhomogeneity of thermal properties. Nevertheless, their incorporation into the geopolymer matrix is also apparent, thereby affirming the efficacy of the fabrication process. Hence, the SEM images confirm not only the existence of PCM capsules within the geopolymer structure but also provide insights into their distribution, size, and quality of binding to the matrix. The acquired micro-photographs corroborate previous findings regarding thermal property measurements, demonstrating that the most uniformly distributed PCM (MikroCaps) contributes to the highest efficiency in terms of insulation and heat accumulation. In the context of engineering applications, these findings highlight that the appropriate selection of PCM type and its effective dispersion within the geopolymer structure are essential for attaining the desired functional properties of the material.

## 4. Discussion

This article presents the findings of a study focused on geopolymer concretes modified with a 15% incorporation of PCMs. The materials MikroCaps, GR42, and PX25 were utilized in this investigation. The analysis encompassed the physical and chemical characteristics of the composites under examination, alongside thermal assessments to ascertain their actual energy efficiency. Furthermore, the study was enhanced by microstructural analysis conducted using scanning electron microscopy. From the standpoint of engineering material development, the integration of PCMs within a geopolymer matrix may signify a significant advancement towards the creation of intelligent composites capable of passively regulating temperature. Research of this nature holds considerable importance in facilitating low-carbon construction and strategies for climate change adaptation.

Each of the PCMs employed contributed to the reduction in the density of the geopolymer samples evaluated, employing both geometric methodologies and calculations based on thermal properties. The most significant decrease in density, amounting to 13%, was noted for the sample incorporating 15 wt.% MikroCaps. The integration of PCMs within the concrete matrix is expected to enhance the density of the resulting composite. Experimental outcomes suggest that the observed decrease in density may be attributable to modifications in the characteristics of the fresh mix, particularly its consistency, which impacts the efficiency of compaction and the dewetting of solids. The results of studies by other authors show that the incorporation of PCM can result in both increases and decreases in the density of geopolymer concrete. Certain studies have demonstrated that the density of geopolymer concrete or mortar marginally increased with the elevation of PCM content utilized as an aggregate [93,94]. In contrast, other investigations have evidenced a decrease in density resulting from the inclusion of PCM in the samples [95,96,97,98]. Notably, the density of samples prepared via impregnation (injection of paraffin into a carrier such as perlite or lightweight aggregates) remained nearly identical to that of the control sample (samples without PCM). This phenomenon can be ascribed to the impregnation of the porosity of the lightweight aggregates by the liquid PCM. Nevertheless, the increase in PCM content corresponded with a reduction in the density of the PCM–geopolymer concrete or mortar, which can be explained by the lower density of MPCM compared to sand and the elevated porosity present in samples containing MPCM.

The analysis of the results concerning compliance with the criteria for the disposal of hazardous waste in specialized landfills indicated that all tested geopolymer samples exhibited leachability levels of individual elements that were significantly lower than the applicable limit standards. Consequently, it can be concluded that these materials do not exhibit the toxic characteristics typical of hazardous waste, rendering them safe from the perspective of environmental protection. Simultaneously, all analyzed samples demonstrated a strongly alkaline reaction (pH > 10), a consequence of the use of alkaline chemical activators during the geopolymer synthesis process. The elevated pH promotes the initiation and progression of the polycondensation reaction, resulting in the formation of a stable and homogeneous binding structure. However, the high alkalinity of the environment may facilitate the release of certain elements, including heavy metals, as observed in the leachability test results, although the levels remained within safe limits. This phenomenon underscores the necessity for additional research into the long-term chemical stability of geopolymers under real-world conditions. Complementing these findings, Nguyen et al. [99] investigated the leachability of heavy metals in geopolymer materials synthesized from red mud and fly ash. Their study revealed that the leachability of components such as Cu and Zn diminished significantly in geopolymer samples compared to raw materials, indicating effective immobilization within the geopolymer matrix. Such results imply that while leachability may pose a concern, well-designed geopolymer mixtures can offer a measure of environmental safety by encapsulating potentially hazardous materials. Additionally, Mashifana and Sillanpää [100] examined the leaching behavior of geopolymers produced from granulated blast furnace slag and fly ash, highlighting apprehensions regarding the leachability of toxic components when these geopolymers come into contact with water. They emphasized that careful formulation and activation methods are essential for minimizing the leaching of harmful substances. This underscores the potential for PCM to either exacerbate or mitigate these issues, depending on their chemical interactions with the geopolymer matrix.

Based on the study of the thermal properties of geopolymer concretes modified with the addition of PCM, it can be seen that each of the components used differentially influenced the key thermal parameters of the composite, including thermal conductivity, specific heat, volumetric specific heat, and thermal diffusivity. The thermal conductivity of the reference sample measured 1.1619 W/m × K. The application of the MikroCaps (MC) additive resulted in a significant decrease of 22.2% in this parameter, indicating a notable reduction in the material’s capacity to transport heat, a phenomenon that may be advantageous in terms of thermal insulation. The incorporation of GR42 led to a reduction in thermal conductivity by 4.6%, whereas PX25, conversely, caused an increase of 13.2%, suggesting that this material possesses a higher intrinsic conductivity or promotes the formation of more compact heat-conducting structures, particularly in a liquid state. The observed reduction in thermal conductivity in samples containing certain PCM additives ought to be interpreted as an indirect effect rather than a direct property of the phase-change material itself. PCM, particularly in microcapsule form, does not definitively exhibit a lower thermal conductivity coefficient compared to other components of the geopolymer matrix. Instead, the decrease in conductivity may result from alterations in the composite’s microstructure, specifically the deterioration of interfacial contact and the reduction in the formation of continuous thermally conductive pathways. Conversely, the increase in thermal conductivity observed with the PX25 additive may be correlated with the development of more compact and well-connected solid phases within the geopolymer concrete structure [101,102]. Hence, the thermal conductivity is contingent not only upon the thermal properties of the PCM itself but also on the modifications in the internal structure of the composite that the PCM indirectly induces. Regarding the material’s capacity to accumulate thermal energy per unit volume, the sample with the PX25 do-additive exhibited a slight increase in volume-specific heat (+0.5%). In contrast, the MC and GR42 additives resulted in a decrease in this parameter by 2.0% and 4.7%, respectively. This indicates that not every PCM exerts a favorable effect on energy storage potential; rather, this effect is significantly influenced by the physicochemical properties of the particular additive. Other authors in various articles have demonstrated that the addition of PCMs in substantial quantities enhances heat capacity [103,104]. The integration of PCM can significantly increase this property, rendering the concrete suitable for thermal energy storage applications [105]. A marked increase in specific heat was observed in samples containing MC (+12.3%) and PX25 (+10.1%), which is a desirable phenomenon in the context of designing materials for passive heat accumulation, as it signifies a greater capacity to absorb thermal energy without a drastic rise in temperature. In contrast, the GR42 sample demonstrated a slight decrease in *C_p_* of 2.1%. As reported in previous studies by the present authors [87,106], it was noted that a high PCM content affects specific heat results positively. The value of thermal diffusivity, which determines the rate of heat propagation within the material, was significantly reduced in the sample containing MC (−20.7%), which may induce a delayed response of the material to temperature variations but simultaneously fosters thermal stabilization. GR42 exhibited a negligible effect on this parameter (<1% change), while PX25 resulted in an increase of 12.4%, which may be advantageous in applications necessitating dynamic thermal energy transfer. The geopolymer concrete showed favorable thermal diffusivity characteristics, likely attributable to its dense matrix and reduced heat transfer due to the incorporation of PCM [107,108].

The results of research concerning geopolymer concretes with the addition of organic PCMs clearly indicate the great potential of these composites in building applications, particularly regarding the enhancement of thermal properties. The incorporation of PCMs enables the development of materials that are capable of storing thermal energy and regulating heat flow, thereby significantly contributing to the energy efficiency of buildings. For instance, samples containing MikroCaps have demonstrated a noteworthy reduction in thermal conductivity, which can be advantageous in thermal insulation applications, while PX25 additives have exhibited a capacity for more dynamic heat exchange, suggesting their utility in partitions that adapt to fluctuating environmental conditions. However, enhancements in thermal performance do not invariably coincide with the preservation of desirable mechanical properties. The observed reduction in density across all samples may indicate a compromise in the material’s structure due to disruptions in the mixture’s rheology and a rise in porosity. These factors may adversely impact mechanical strength, a critical limitation in the design of structural components, especially those that bear loads. Consequently, a classic trade-off arises: the augmentation of thermal energy storage capacity might impose a detriment to the stiffness or mechanical durability of the composite. Practical applications must, therefore, consider the functional context of a given structural element. PCM geopolymer composites exhibiting low thermal conductivity and high specific heat are likely to excel as insulation or accumulation elements within the building envelope. In such instances, a slight reduction in strength may be deemed acceptable. On the other hand, materials with increased thermal conductivity and diffusivity, including those containing PX25 additives, can be advantageous in active heat management systems, such as in underfloor heating or technical enclosures. The advancement of geopolymers containing PCM additives paves the way for new design possibilities; however, their implementation necessitates a harmonious balance between thermal and mechanical properties. The selection of a specific PCM should always be preceded by a thorough analysis of performance requirements. Future research ought to concentrate on the long-term thermal–mechanical stability and behavior of the composites under real-world conditions. Only in this manner can we fully realize their potential in contemporary, energy-efficient construction.

## 5. Conclusions

Based on the preceding discussion regarding the results of the study that examined the physical, chemical, and thermal properties of geopolymer concretes modified by the inclusion of PCMs, the ensuing post-summary conclusions may be articulated as follows:

1. The introduction of various types of PCMs (MikroCaps, GR42, PX25) affected the physicochemical and thermal properties of geopolymers in different ways. This observation confirms that the characteristics of a specific PCM are crucial in shaping the final parameters of the composite.
2. The addition of PCM reduced the density of the composites. This phenomenon can be attributed to the modification of the rheological properties of the mixture and changes in the degree of density and distribution of solid components.
3. All samples satisfied the criteria for non-hazardous waste disposal, demonstrating levels of elemental leachability significantly below acceptable standards. At the same time, the strongly alkaline reaction of the mixtures (pH > 10) facilitated polycondensation processes; however, it might also influence the mobility of certain ions, including heavy metals.
4. PCMs affected thermal conductivity to varying extents. The most significant decrease in thermal conductivity was noted for MikroCaps, with a reduction of 22.2%, whereas PX25 resulted in an increase of 13.2%. This observation suggests that the thermal conductivity of the material is indirectly dependent on its microstructure and porosity, rather than being solely contingent upon the conductivity of the PCM.
5. The observed increase in specific heat for certain samples (MC, PX25) suggests their potential for thermal energy storage. At the same time, the changes in specific heat by volume remained non-negligible, indicating the limited effect of PCMs on their long-term energy storage capacity per unit volume.
6. The parameter of thermal diffusivity exhibited the most significant changes in the samples with MikroCaps (−20.7%) and PX25 (+12.4%). This observation leads to the conclusion that different PCMs can be utilized for both thermal insulation, characterized by slower heat transport, and dynamic thermal control, which is marked by faster conduction.
7. The selection of a suitable PCM should be subject to specific functional requirements, whether the objective is to enhance thermal insulation, augment thermal capacity, or dynamically respond to fluctuations in ambient temperature. The findings of the study reveal significant potential for the incorporation of geopolymers with PCM additives in next-generation construction materials, particularly in relation to sustainability and energy efficiency.

The use of PCMs in geopolymer concretes enables effective alterations to their thermal and physical properties while maintaining a significant standard of environmental safety. The differences in results among various PCM additives highlight the need for careful selection concerning their intended applications, such as thermal insulation, energy accumulation, or dynamic responsiveness to temperature fluctuations. These findings provide a strong foundation for developing modern building materials based on geopolymer technology, incorporating PCM additives.

The research findings confirm that PCM additives significantly alter the characteristics of geopolymer concrete, thus facilitating the design of materials with specific physical-thermal properties. The results presented constitute a substantial contribution to the advancement of sustainable construction technologies, integrating the principles of energy efficiency, durability, and environmental sustainability. Future research endeavors should encompass an examination of the long-term thermal and mechanical stability of these composites, in addition to their performance under operational conditions, which will facilitate a comprehensive evaluation of their practical utility at the engineering scale.

## Figures and Tables

**Table 1:** Oxide analysis for raw materials.

Precursor	Oxide Composition (wt.%)									
	SiO_2_	Al_2_O_3_	Fe_2_O_3_	CaO	K_2_O	TiO_2_	SO_3_	Br	CuO	OsO_4_
Fly ash	59.21	31.05	3.88	2.29	2.07	0.77	0.50	-	-	-
Sand	98.57	-	0.30	0.36	0.42	-	0.24	-	-	-
Cement	-	82.85	-	17.09	-	-	-	-	-	-
Microspheres	55.38	38.22	2.44	0.67	2.15	0.85	0.12	-	-	-

**Table 2:** Particle size analysis of raw materials.

Material	D_10_ [mm]	D_50_ [mm]	D_90_ [mm]	Average Value [mm]	Standard Deviation [mm]
Fly ash	2.45	12.87	32.12	16.21	0.03
Sand	253.72	341.97	472.38	390.65	0.02
Cement	1.62	11.39	29.90	14.70	0.01
Microspheres	18.93	53.39	88.89	56.39	0.07
Syringaldehyde	3.04	3.78	7.24	5.07	0.21

**Table 3:** Particle size analysis of PCMs.

Material	D_10_ [mm]	D_50_ [mm]	D_90_ [mm]	Average Value [mm]	Standard Deviation [mm]
MikroCaps	2.23	5.85	9.48	6.25	0.03
GR42	34.21	104.73	138.49	103.10	0.05
PX25	3.06	10.19	21.64	11.99	0.04

**Table 4:** Density *ρ*_b1_ and *ρ*_b2_ of geopolymer concretes with PCMs.

Designation of Samples	F.A.—ref.	15 wt.% MC	15 wt.% GR42	15 wt.% PX25
*ρ*_b1_ [kg/m^3^]	1832.28	1601.60	1780.30	1671.55
*ρ*_b2_ [kg/m^3^]	1834.88	1602.65	1785.30	1675.90

**Table 5:** Analysis of concentrations of harmful substances in geopolymer concretes with PCMs.

Permissible Leaching Limits *				F.A. —ref.	15 wt.% MC
Liquid/Solid Phase = 10 L/kg [mg/kg Dry Weight] Baseline Test				mg/kg	mg/kg
Component	Criteria for Allowing Inert Waste to Be Deposited in an Inert Waste Landfill	Criteria for Allowing Hazardous Waste to Be Disposed of in a Hazardous Waste Landfill	Criteria for Allowing Non-Hazardous and Inert Waste to Be Deposited in a Landfill for Non-Hazardous and Inert Waste		
Arsen (As)	0.5	25	2	5.5	3.3
Bar (Ba)	20	300	100	0.25	0.53
Cadmium (Cd)	0.04	5	1	<0.0020	<0.0020
Total chromium (Cr)	0.5	70	10	0.087	0.083
Copper (Cu)	2	100	50	0.22	0.37
Mercury (Hg)	0.01	2	0.2	<0.010	<0.010
Molybdenum (Mo)	0.5	30	10	3.0	1.5
Nickel (Ni)	0.4	40	10	0.028	0.028
Lead (Pb)	0.5	50	10	<0.020	0.020
Antimony (Sb)	0.06	5	0.7	<0.020	<0.020
Selen (Se)	0.1	7	0.5	0.55	0.91
Zinc (Zn)	4	200	50	0.079	0.17
Chlorides (Cl^−^)	800	25,000	15,000	130	130
Fluorides (F^−^)	10	500	150	21	22
Sulfates (SO_4_^2−^)	1000	50,000	20,000	2800	2900
Dissolved organic carbon (DOC)	500	1000	800	80	1000
Dissolved solids (TDS **)	4000	100,000	60,000	44,000	32,000
Chromium (VI) (Cr^6+^)				22	35
pH				10.6	10.6

(*) Permissible leaching limits for waste disposed of in landfills equipped with leachate collection systems subsequently directed to wastewater treatment plants, except for DOC and TDS components, which are considered to be met for values higher than those specified in the table. (**) Values for dissolved solid compounds (TDS) can be used interchangeably for sulfate and chloride values.

**Table 6:** Thermal parameters for geopolymer concretes with PCMs.

Statistical Parameters	Thermal Properties of Sample F.A.—ref.			
	*λ* [W/(m × K)]	*C_v_* [MJ/(m^3^ × K)]	*C_p_* [J/(kg × K)]	*α* [mm^2^/s]
Quartile, *Q1*	1.1582	1.8120	987.9	0.6379
Median, *M*	1.1616	1.8145	989.3	0.6403
Quartile, *Q3*	1.1646	1.8170	990.7	0.6422
Interquartile range, *IQR = (Q3 − Q1)*	0.0065	0.0050	2.7	0.0043
Higher outlier, *HO = Q3 + 1.5·IQR*	1.1743	1.8245	994.7	0.6486
Lower outlier, *LO = Q1 − 1.5·IQR*	1.1485	1.8046	983.9	0.6314
**Average value,** $\bar{X}$	**1.1619**	**1.8141**	**989.1**	**0.6403**
**Standard deviation, *s***	**0.0047**	**0.0036**	**2.0**	**0.0027**
Coefficient of variation, *CV* [%]	0.40	0.16	0.20	0.42
Upper critical value, *UCV*	1.1671	1.8179	991.2	0.6431
Lower critical value, *LCV*	1.1566	1.8103	987.1	0.6375
**Statistical parameters**	**Thermal properties of sample 15 wt.% MC**			
	***λ*** **[** **W/(m × K)]**	***C_v_*** **[** **MJ/(m** **^3^ × K)]**	***C_p_*** **[** **J/(kg × K)]**	***α*** **[mm^2^/s]**
Quartile, *Q1*	0.8642	1.7696	1104.9	0.4838
Median, *M*	0.8848	1.7784	1110.4	0.4971
Quartile, *Q3*	0.9455	1.7882	1116.6	0.5304
Interquartile range, *IQR = (Q3 − Q1)*	0.0813	0.0186	11.7	0.0466
Higher outlier, *HO = Q3 + 1.5·IQR*	1.0674	1.8161	1134.1	0.6004
Lower outlier, *LO = Q1 − 1.5·IQR*	0.7424	1.7417	1087.4	0.4139
**Average value,** $\bar{X}$	**0.9037**	**1.7786**	**1110.6**	**0.5080**
**Standard deviation, *s***	**0.0760**	**0.0131**	**8.2**	**0.0416**
Coefficient of variation, *CV* [%]	8.18	0.73	0.73	8.00
Upper critical value, *UCV*	0.9897	1.7924	1119.2	0.5517
Lower critical value, *LCV*	0.8175	1.7648	1102.0	0.4643
**Statistical parameters**	**Thermal properties of sample 15 wt.% GR42**			
	***λ*** **[** **W/(m × K)]**	***C_v_*** **[** **MJ/(m** **^3^ × K)]**	***C_p_*** **[** **J/(kg × K)]**	***α*** **[mm^2^/s]**
Quartile, *Q1*	1.1042	1.7270	967.7	0.6392
Median, *M*	1.1100	1.7288	968.6	0.6424
Quartile, *Q3*	1.1139	1.7300	969.4	0.6450
Interquartile range, *IQR = (Q3 − Q1)*	0.0097	0.0031	1.7	0.0058
Higher outlier, *HO = Q3 + 1.5·IQR*	1.1284	1.7347	971.9	0.6537
Lower outlier, *LO = Q1 − 1.5·IQR*	1.0897	1.7224	965.1	0.6306
**Average value,** $\bar{X}$	**1.1090**	**1.7284**	**968.4**	**0.6418**
**Standard deviation, *s***	**0.0070**	**0.0023**	**1.3**	**0.0043**
Coefficient of variation, *CV* [%]	0.65	0.13	0.13	0.67
Upper critical value, *UCV*	1.1169	1.7308	969.8	0.6463
Lower critical value, *LCV*	1.1011	1.7260	967.1	0.6373
**Statistical parameters**	**Thermal properties of sample 15 wt.% PX25**			
	***λ*** **[** **W/(m × K)]**	***C_v_*** **[** **MJ/(m** **^3^ × K)]**	***C_p_*** **[** **J/(kg × K)]**	***α*** **[mm^2^/s]**
Quartile, *Q1*	1.2130	1.8156	1084.2	0.6658
Median, *M*	1.3100	1.8219	1088.0	0.7178
Quartile, *Q3*	1.3963	1.8325	1094.4	0.7613
Interquartile range, *IQR = (Q3 − Q1)*	0.1834	0.0170	10.2	0.0955
Higher outlier, *HO = Q3 + 1.5·IQR*	1.6714	1.8580	1109.7	0.9046
Lower outlier, *LO = Q1 − 1.5·IQR*	0.9380	1.7902	1068.9	0.5225
**Average value,** $\bar{X}$	**1.3148**	**1.8238**	**1089.1**	**0.7195**
**Standard deviation, *s***	**0.1057**	**0.0090**	**5.4**	**0.0553**
Coefficient of variation, *CV* [%]	7.85	0.49	0.49	7.55
Upper critical value, *UCV*	1.4346	1.8332	1094.8	0.7775
Lower critical value, *LCV*	1.1949	1.8144	1083.5	0.6615

**Table 7:** Mean values of thermal parameters for geopolymer concretes with PCMs.

ID	Thermal Properties of Geopolymer Concretes with PCMs			
	*λ* [W/(m × K)]	*C_v_* [MJ/(m^3^ × K)]	*C_p_* [J/(kg × K)]	*α* [mm^2^/s]
F.A.—ref.	1.1619 ± 0.0047	1.8141 ± 0.0036	989.1 ± 2.0	0.6403 ± 0.0027
15 wt.% MC	0.9037 ± 0.0760	1.7786 ± 0.0131	1110.6 ± 8.2	0.5080 ± 0.0416
15 wt.% GR42	1.1090 ± 0.0070	1.7284 ± 0.0023	968.4 ± 1.3	0.6418 ± 0.0043
15 wt.% PX25	1.3148 ± 0.1057	1.8238 ± 0.0090	1089.1 ± 5.4	0.7195 ± 0.0553

**Table 8:** Confidence intervals of measured thermal properties of geopolymer concretes with PCMs.

Test Sample	Determined Confidence Intervals
F.A.—ref.	*p* (1.1566≤ *λ* ≤ 1.1671) = 0.95
	*p* (987.1 ≤ *C_p_* ≤ 991.2) = 0.95
	*p* (0.6375≤ *α* ≤ 0.6431) = 0.95
15 wt.% MC	*p* (0.8175 ≤ *λ* ≤ 0.9897) = 0.95
	*p* (1102.0 ≤ *C_p_* ≤ 1119.2) = 0.95
	*p* (0.4643 ≤ *α* ≤ 0.5517) = 0.95
15 wt.% GR42	*p* (1.1011 ≤ *λ* ≤ 1.1169) = 0.95
	*p* (967.1 ≤ *C_p_* ≤ 969.8) = 0.95
	*p* (0.6373 ≤ *α* ≤ 0.6463) = 0.95
15 wt.% PX25	*p* (1.1949≤ *λ* ≤ 1.4346) = 0.95
	*p* (1083.5 ≤ *C_p_* ≤ 1094.8) = 0.95
	*p* (0.6615 ≤ *α* ≤ 0.7775) = 0.95

## Data Availability

The original contributions presented in this study are included in the article. Further inquiries can be directed to the corresponding authors.
